# Identification of a Core Bacterial Community within the Large Intestine of the Horse

**DOI:** 10.1371/journal.pone.0077660

**Published:** 2013-10-24

**Authors:** Kirsty Dougal, Gabriel de la Fuente, Patricia A. Harris, Susan E. Girdwood, Eric Pinloche, C. Jamie Newbold

**Affiliations:** 1 Institute of Biological Environmental and Rural Sciences, Aberystwyth University, Aberystwyth, Ceredigion, United Kingdom; 2 Equine Studies Group, WALTHAM Centre for Pet Nutrition, Leicestershire, United Kingdom; Rockefeller University, United States of America

## Abstract

The horse has a rich and complex microbial community within its gastrointestinal tract that plays a central role in both health and disease. The horse receives much of its dietary energy through microbial hydrolysis and fermentation of fiber predominantly in the large intestine/hindgut. The presence of a possible core bacterial community in the equine large intestine was investigated in this study. Samples were taken from the terminal ileum and 7 regions of the large intestine from ten animals, DNA extracted and the V1-V2 regions of 16SrDNA 454-pyrosequenced. A specific group of OTUs clustered in all ileal samples and a distinct and different signature existed for the proximal regions of the large intestine and the distal regions. A core group of bacterial families were identified in all gut regions with clear differences shown between the ileum and the various large intestine regions. The core in the ileum accounted for 32% of all sequences and comprised of only seven OTUs of varying abundance; the core in the large intestine was much smaller (5-15% of all sequences) with a much larger number of OTUs present but in low abundance. The most abundant member of the core community in the ileum was *Lactobacillaceae*, in the proximal large intestine the *Lachnospiraceae* and in the distal large intestine the *Prevotellaceae*. In conclusion, the presence of a core bacterial community in the large intestine of the horse that is made up of many low abundance OTUs may explain in part the susceptibility of horses to digestive upset.

## Introduction

The presence of a core microbiome in the gut (i.e. key microbes that are present in all or the majority of individuals within a population) has been much debated [1-3]. It has been suggested that if a core community could be identified across all healthy members of a species this may provide a basis for disease diagnosis, prevention and possibly provide therapeutic targets [4,5]. Since the concept was initially posed there has been speculation as to whether a core may be gene or functionally based [1,6]; and while an apparent core microbiome in the human gut has been demonstrated in several studies no clear definition of what constitutes a core and how large the core is in any community exists [3].

The human gut microbiota appears to be relatively stable in composition over time, with individuals maintaining a similar microbial profile whilst on a uniform diet with no gastrointestinal upset [7,8]. In horses it is apparent that the microbiome of the hind gut changes rapidly in early life from birth up until 56 days of age [9] and even in adult animals may not be stable. Indeed recent work using terminal restriction fragment length polymorphism demonstrated a statistically significant (P<0.001) bacterial community in the gut after a ten week period in six ponies fed a constant high fibre based diet [10]. This would suggest that the equine gut microbial community may not be as stable as that found in the human gut. The horse receives much of its dietary energy through microbial hydrolysis and fermentation of fibre predominantly in the large intestine/hindgut [11] as the microbiota ferment fibre to yield volatile fatty acids which can be absorbed and utilised as energy sources [12-14]. 

Horses are however extremely susceptible to gastrointestinal disturbance when subjected to sudden changes in diet which can result in alteration of fermentation patterns and ultimately metabolic disorder. Colic and laminitis commonly occur with colic being the biggest cause of mortality in the horse [15,16]. Colic occurs in almost 6% of thoroughbred training facilities in the UK [17] and is the cause of almost 15% of equid deaths in the USA [18]. The prevalence of laminitis in the UK is poorly documented but between 0.5 and 3% of the equid population has been reported as suffering from the condition [19,20]. When horses are challenged by the sudden incorporation of high levels of soluble carbohydrate in the diet (e.g. starch from grain or starch/simple sugars/fructan from pasture) normal enzymatic breakdown in the small intestine is overloaded and large amounts of readily fermentable starch/sugar pass to the large intestine where microbial fermentation occurs [21-23]. Rapid fermentation occurs with a rapid accumulation of lactic acid producing bacteria such as *Lactobacilli* and *Streptococci* occurring [21,22,24,25]. High amounts of lactic acid and gas are produced causing a rapid drop in hindgut pH and death/ suppression of the normal fiber degrading bacteria. These events are thought to contribute in some instances to impaction colic due to excess gas accumulation combined with dehydration of digesta [21,26] and may also help to explain some instances of laminitis [25,27]. 

Recently it has been suggested that microbial factors might influence the development and progression of chronic laminitis and colic [28,29]. In particular Steelman and colleagues [28] found a higher bacterial diversity in horses with chronic laminitis but it is not clear if this represented a less stable microbiota in these animals. Very little attention however, has been given to the question of whether horses have a core gut microbiome, although the recent publication by Costa and colleagues [29] found common operational taxonomic units (OTUs) across several horses. Here we have investigated the possibility that either 1) the horse has an intrinsically unstable gut microbial fermentation because it has an unusual core bacterial community that is large and dependent on only a few key bacterial phyla or 2) alternatively there is no core or a core made up of many low abundance species leading to an intrinsically unstable bacterial community in the equine hindgut.

## Materials and Methods

### Collection of gut samples

Procedures carried out as part of this research did not constitute an experiment as defined under the Animals (Scientific Procedures) Act 1986. However, all evaluations and procedures were carried out following authorisation by Aberystwyth University’s local Ethical Review Committee. Samples were obtained from animals euthanized for non-research purposes at LJ Potters, Taunton, Somerset abattoir and all were stunned before slaughter for meat in accordance with Welfare of Animals (Slaughter or Killing) Regulations 1995. There were five Thoroughbred horses (Ages 5, 7, 8, 8 and 9 years old) and five British native breed ponies (Ages 7, 8, 13, 17 and 19 years old). Pre-euthanasia the horses and ponies had all been maintained on a grass based diet but were fed hay for 12 hours pre-euthanasia. Although the change to hay at this time may have had a small effect on the microflora of the hind gut all animals received the same dietary treatment. The ponies came from the same New Forest area in the UK (see Table S1), therefore were likely on similar diets, while the Thoroughbred horses came from one location grazing a single grass pasture. Based on owner provided information (see Table S1) none of the animals had suffered from episodes of metabolic disease (including laminitis) or intestinal disturbances (including colic) within the previous 6 months. 

Samples of luminal gut contents were collected by the authors between 5 and 10 minutes after euthanasia from the terminal ileum and seven regions of the large intestine (caecum, right ventral colon (RVC), left ventral colon (LVC), left dorsal colon (LDC), right dorsal colon (RDC), small colon (SC) and faeces from the rectum). Each region of the large intestine was identified then sampled in a consistent manner according to the sequence listed above. For each region complete contents were removed into a clean bucket, mixed thoroughly and then sub sampled into grip top bags (in triplicate).

Samples were then placed on ice before being transferred on dry ice for transportation back to the laboratory (time lag of around 4 hours) and finally stored at -80°C until required for analysis. For all downstream methods each of the triplicate subsamples for each region was processed individually.

### DNA extraction

Prior to extraction of nucleic acids, samples were freeze dried then disrupted by bead beating. Freeze- dried samples (100mg) were added to a 2ml screw top tube and then one autoclaved glass ball added (4mm, undrilled, G/0300/53, Fisher Scientific, UK). Samples were beaten for 90s at 5000 rpm (maximum speed) in a Mini-Beadbeater™ (Biospec products Inc., Bartlesville, OK). DNA was then extracted using QIAGEN QIAamp® DNA stool mini kits (Qiagen Ltd., UK) using the method described by Skřivanová and colleagues [30]. 

### PCR amplification of 16S rRNA

Amplification of the V1-V2 hyper variable regions of 16S rRNA was carried out with primers 27F and 357R [31]. The forward primer (5’-AGAGTTTGATCMTGGCTCAG-3’) carried the 454 Lib-L adaptor sequence B (5’-CCTATCCCCTGTGTGCCTTGGCAGTCTCAG-3’) and the reverse primer (5’-ACGAGTGCGTCTGCTGCCTYCCGTA-3’) carried the 454 Lib-L adaptor sequence A (5’-CCATCTCATCCCTGCGTGTCTCCGACTCAG-3’) followed by a 10 nucleotide sample specific barcode sequence (See Table S2). For each sample replicate PCR was performed in duplicate ; a 25µl reaction was prepared containing 5U µl ^-1^ FastStart High Fidelity Enzyme Blend, 10x FastStart High Fidelity Buffer with 18mM MgCl_2_(Roche Diagnostics Ltd., Burgess Hill, UK), 0.2mM of each dNTP (Promega UK Ltd. Southampton, UK) with each primer used at 0.2µM. For each reaction 1µl DNA template at 2.5-125ng/µl (as per Roche FastStart high Fidelity system recommendations) was used. The conditions used were a hot start of 95°C for 10 min, 95°C for 2 min followed by 22 cycles of 95°C for 30s, 60°C for 30s and 72°C for 45s with a final extension at 72°C for 7 min. Reactions were amplified in a T100™ thermal cycler (Bio-Rad, Hemel Hempstead, UK). Resultant amplicons were visualized on a 1% (w/v) TAE agarose gel to assess quality of amplification before pooling the duplicate reactions.

### Short fragment removal and pooling of libraries and sequencing

Pooled PCR reactions for all sample replicates were purified as per Roche technical bulletin 2011-007 (January 2012) ‘Short Fragment Removal Procedure for the Amplicon Library Preparation Procedure’ using Agencout AMpure XP beads (Beckman Coulter Inc.,Fullerton, USA). DNA concentration of the purified PCR products was assessed using an Epoch Microplate Spectrophotometer with a Take3 Micro-Volume plate (BioTek UK, Potton, UK) to enable equi-molar pooling of samples into six libraries containing forty samples with unique barcode sequences. Each library was further purified using the E-Gel® System with E-Gel® SizeSelect™ 2% Agarose gel (Life Technologies Ltd, Paisley, UK). A final purification step using Agencout AMpure XP beads standard PCR purification procedure (Beckman Coulter Inc.,Fullerton, USA) was carried out for each library. To assess the purity of the sample libraries a quality control PCR was carried out for each as detailed in Roche technical bulletin 2011-007. 25µl reactions were prepared containing: 5U µl ^-1^ FastStart High Fidelity Enzyme Blend, 10x FastStart High Fidelity Buffer with 18mM MgCl_2_ (Roche Diagnostics Ltd., Burgess Hill, UK), 0.2mM of each dNTP (Promega UK Ltd. Southampton, UK) with each primer used at 0.2µM. Primers used were the same as the Lib-L adapter sequences described previously as recommended in the Roche Technical Bulletin 2011-007. For each reaction 1µl of each library containing 2x10^8^ molecules/µl was used. The conditions used were 94°C for 11 min followed by 20 cycles of 94°C for 1min, 60°C for 1min and 72°C for 1min with a final extension at 72°C for 10 min. On completion PCR products were incubated for 30min at 37°C with 0.5µl of Exonuclease I (New England BioLabs (UK) Ltd. Hitchin, UK). Reactions were amplified in a T100™ thermal cycler (Bio-Rad, Hemel Hempstead, UK). Products from the quality control PCR were assessed for quality and purified libraries were quantified on an Agilent 2100 Bioanalyzer with a High Sensitivity DNA chip (Agilent Technologies UK Ltd, Stockport, UK). The sample libraries were subsequently sequenced using the Roche 454 GS FLX Titanium series sequencer following ‘emPCR Method Manual-Lib-L’.

### Sequence filtering, processing and statistical analysis

Following sequencing data was combined and sample id’s assigned to multiplexed reads using the MOTHUR software environment [32]. Sequence data was denoised, low quality sequences, pyrosequencing errors and chimeras were removed then sequences were clustered into OTUs at 97% identity using the CD-HIT-OTU pipeline (available from http://eeizhong-lab.ucsd.edu/cd-hit-otu) [33]. OTUs containing fewer than four reads per gut site in any individual animal were excluded due to the likelihood of them being a sequencing artefact. Samples were normalised by randomly resampling across all samples such that the number of sequences per gut site was equal to the lowest number seen (3225 sequences per sample which gave 32250 per gut region when all animals were combined) using Daisychopper (www.genomics.ceh.ac.uk/GeneSwytch/). Taxonomic classification of OTUs was carried out using the Ribosomal Database Project (RDP) Classifier [34]. 

Data was prepared and tables and figures produced using Microsoft Excel and the ‘R’ software environment (version 2.15; http//www.r-project.org/).Simpson and Shannon-Wiener diversity indices were calculated using normalised data as recommended to reduce over inflation of true diversity in pyrosequencing data sets [35]. Species richness, diversity were then analysed by two-way ANOVA using GenStat® 12th edition.The core community at OTU level in any gut region was defined by being present in all of the ten animals included in the study. 

### Nucleotide sequence accession numbers

16S rRNA sequences were deposited with the EBI Sequence Read Archive (SRA) under study accession number: ERP002202.

### Absolute 16S rRNA quantification

Total bacteria were quantified by quantitative PCR (q-PCR) using specific 16S rRNA targeted primers forward-GTGSTGCAYGGYTGTCGTCA reverse- ACGTCRTCCMCACCTTCCTC [36] as described in previous work [37] but using the reaction mix of 3pmol of each primer and 5µl of SYBR® Green JumpStart™ Taq ReadyMix™ (Sigma-Aldrich, Dorset, UK) with a total reaction volume of 10µl. For cycling a Roche LightCycler® 480 II was used and data analysed using LightCycler® 480 software (version 1.5.0.39) (Roche Diagnostics Ltd, Burgess Hill, UK). The preparation for sequencing q-PCR was conducted on each of the triplicate subsamples with the data presented showing the mean result. Data was then analysed by two-way ANOVA considering gut region and animal using GenStat® 12th edition.

## Results

One million, four hundred and sixty thousand, one hundred and twenty four sequences of average length 358bp were obtained from the 454 FLX Titanium sequencing. Quality filtering resulted in 559 623 high quality sequences providing 32 250 sequences per gut region after normalisation. Sequences were clustered into 4818 unique OTUs across the complete data set. Rarefaction curves (which were calculated from non-normalised data, Figure S1) showed that for each gut region curves had not plateaued indicating that complete sampling of these environments had not yet been achieved. Good’s coverage estimates, however, indicate that a large part of the diversity in all regions had been captured with the average coverage by gut region being 95.7% (s.d. 0.9) and by animal also 95.7% (s.d. 1.0) (Figures S2 & S3).

The bacterial community within the different regions of the horse gut was found to be highly diverse and even (as indicated by the Simpson and Shannon-Weiner diversity indices, see Table 1) with significant differences between gut regions (P<0.001). The microbiota of the ileum was significantly less diverse than that of any region of the large intestine, with little variation between the different large intestine regions. Similarly the species richness (number of OTUs) and bacterial load (determined by q-PCR) was lowest in the ileum. Although not significantly different across the large intestine regions, species richness was highest in the RVC, with the quantity of DNA increasing progressively through the intestinal tract, being highest in the faeces. 

**Table 1:** Diversity and Richness of the bacterial communities in different compartments of the horse’s large intestine.

	Ileum	Caecum	Right ventral colon	Left ventral colon	Left dorsal colon	Right dorsal colon	Small colon	Faeces	LSD	P value
Species Richness	281^a^	667^b^	707^b^	677^b^	668^b^	652^b^	631^b^	631^b^	95.1	<0.001
Simpson	0.930^a^	0.993^b^	0.994^b^	0.993^b^	0.988^b^	0.988^b^	0.985^b^	0.985^b^	0.0154	<0.001
Shannon	3.74^a^	5.76^bc^	5.87^c^	5.76^bc^	5.58^bc^	5.52^bc^	5.42^b^	5.40^b^	0.366	<0.001
q-PCR (ng DNA/mg DM)	235^a^	355^a^	451^a^	876^b^	871^b^	1008^b^	1034^b^	1600^c^	368	<0.001

Different superscript letters denote significant differences.

**Figure 1:**
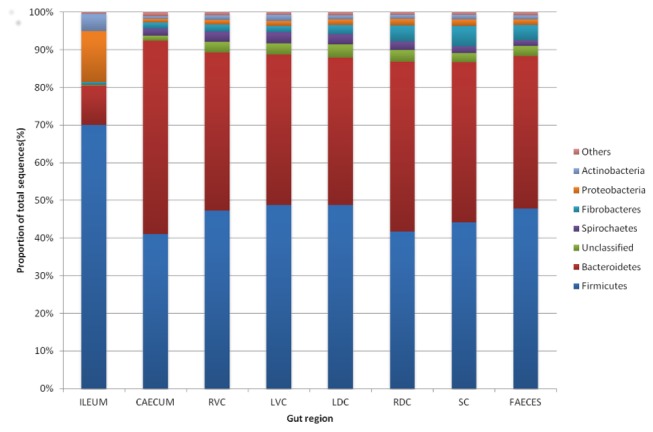
Phyla identified and relative proportion of each in different compartments of the horse’s large intestine. Data shown from the Ileum, caecum, right ventral colon (RVC), left ventral colon (LVC), left dorsal colon (LDC), right dorsal colon (RDC), small colon and faeces. The category others includes; *TM7, Tenericutes, synergistetes, Cyanobacteria/Chloroplast, SR1, Elusimicrobia, Fusobacteria, Chloroflexi*.

**Figure 2:**
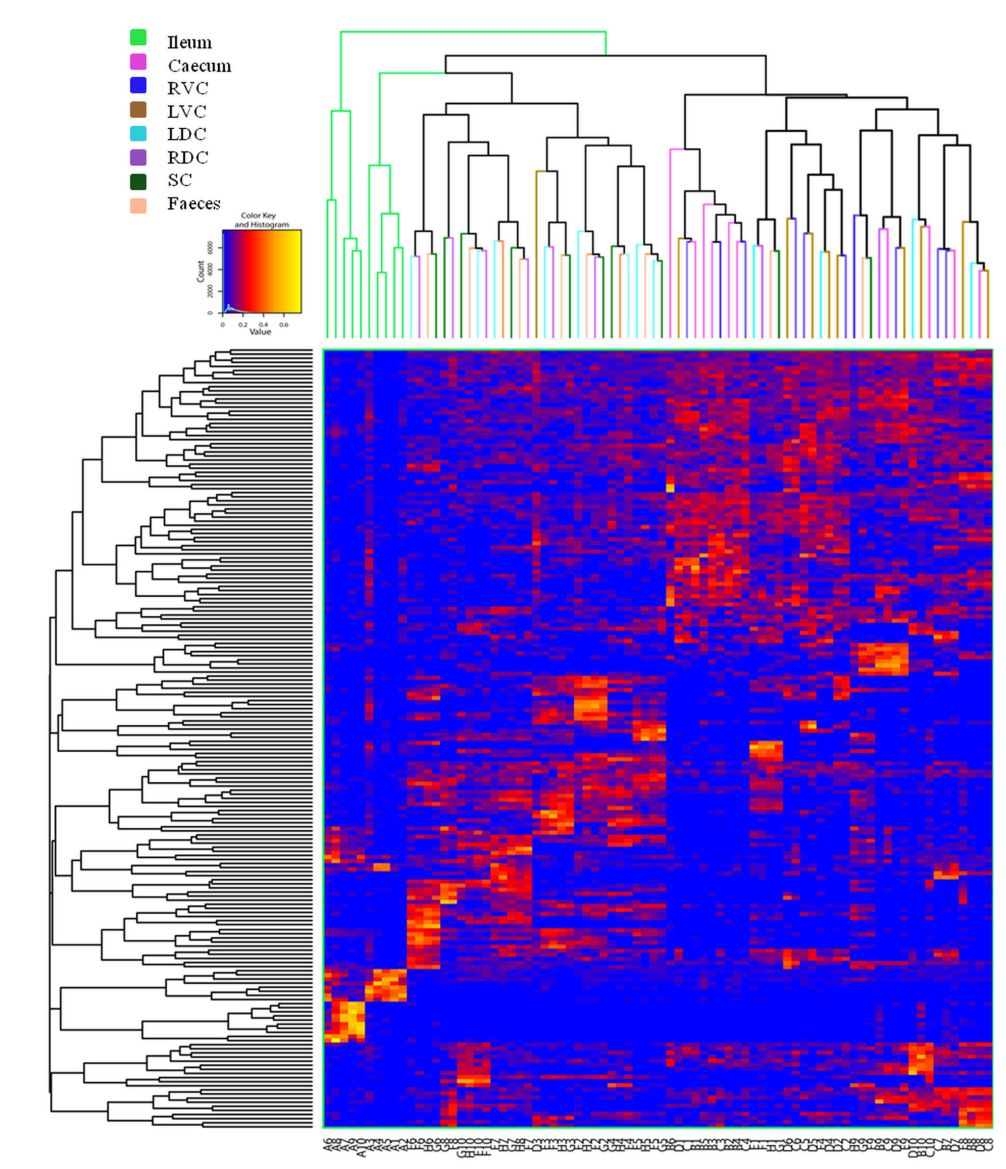
Heatmap showing OTUs in different compartments of the horse’s large intestine. Data shown from the ileum (A) caecum (B), right ventral colon (C), left ventral colon (D), left dorsal colon (E), right dorsal colon (F), small colon (G) and faeces (H) found at 0.1% or greater abundance as a percentage of the total number of sequences (accounts for 49.28% of the total sequences). The cladogram on the vertical axis shows the phylogenetic relationship between the individual OTUs included in this analysis. The cladogram on the horizontal axis shows the phylogentic relationship between all the OTUs identified in each sample.

Based on classification of recovered OTUs, differences between the ileum and the large intestine can be explained by the most abundant phyla (Figure 1). In all regions of the large intestine the most dominant phyla were the *Firmicutes* (46%) and *Bacteroidetes* (43%). In the ileum the *Firmicutes* (70%) were the most dominant followed by *Proteobacteria* (14%) then *Bacteroidetes* (10%). The pattern of phyla seen in Figure 1 shows a high degree of similarity throughout the large intestine. Figure 2 shows a 2D heatmap of OTUs present at 0.1% or more of the total number of sequences across all samples, clustered by sample as well as by OTU. It can be clearly seen that a specific group of OTUs cluster in all ileal samples and a distinct and different signature exists for the proximal regions of the large intestine (caecum, RVC & LVC) and the distal regions (RDC, SC & faeces) with the LDC appearing in both groups. Although the regions of the gut cluster as detailed there also appears to be a large amount of inter-animal variation within these groupings.

**Figure 3:**
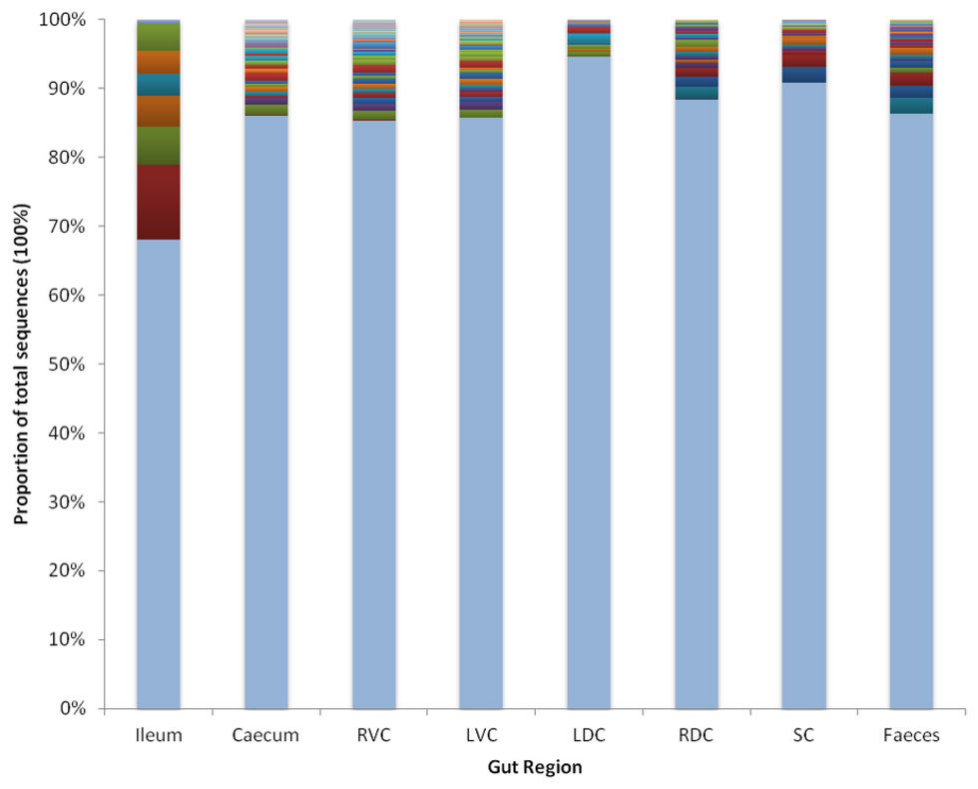
The core bacterial community in different compartments of the horse’s large intestine. The core community is defined as those OTUs (clustered at 97% similarity) present in all ten animals and which abundances are 0.1% (or greater) of the total number of sequences in that gut region. The lower pale blue section of the bar indicates the proportion that is not part of the core. The remaining individual coloured sections represent each OTU of which the core is comprised; Ileum (7), caecum (31), right ventral colon (RVC) (33), left ventral colon (LVC) (28), left dorsal colon (LDC) (12), right dorsal colon (RDC) (18), small colon (16) and faeces (25). For further details see table S3.

In every gut region a number of OTUs have been identified which make up the core community (found in all animals). To account for ambiguity surrounding the definition of bacterial classification OTUs were clustered at 90%, 95%, 97% and 99% similarity (Table 2) allowing the size of the core community to be shown in terms of a percentage of the total number of OTUs identified at each level of similarity. As the clustering threshold increases the total number of OTUs identified increases and as such the size of the core decreases. At all levels of clustering similarity the core community is smallest in the ileum and largest in the caecum. When taking account not only of the number of OTUs making up the core but also the number of occurrences within each OTU a different impression of the core microbiota can be seen (Figure 3, OTUs clustered at 97% similarity and present at 0.1% of community or greater). As with all previous data the community within the ileum is different from that of the large intestine having a core made up of approximately 32% of all sequences but comprised of only seven OTUs of varying abundance, including one OTU responsible for 11% of all sequences. The core in the large intestine, however, is much smaller (5-15% of the total number of sequences) and shows a different pattern to that of the ileum with a much larger number of OTUs being present but in low abundance (the largest single OTU in any region of the large intestine accounts for only 2% of all sequences for that region).

**Table 2:** Size of the core community in different compartments of the horse’s large intestine.

	ILEUM	CAECUM	RVC	LVC		LDC	RDC	SC	FAECES
90%	10/571	75/599	85/681	72/666		57/795	70/760	55/746	62/728
	(1.8%)	(12.5%)	(12.5%)	(10.8%)		(7.2%)	(9.2%)	(7.4%)	(8.5%)
95%	8/1154	62/1517	64/1741	52/1730	29/2013		36/1895	36/1843	38/1844
	(0.7%)	(4.1%)	(3.7%)	(3%)		(1.4%)	(1.9%)	(2%)	(2.1%)
97%	7/1478	31/2263	33/2526	28/2480		12/2802	19/2683	16/2520	25/2566
	(0.5%)	(1.4%)	(1.3%)	(1.1%)		(0.4%)	(0.7%)	(0.6%)	(1%)
99%	8/1980	62/3747	64/4050	52/3860		30/4145	36/3823	36/3731	38/3722
	(0.4%)	(1.7%)	(1.6%)	(1.3%)		(0.7%)	(0.9%)	(1%)	(1%)

Data shown from the Ileum, caecum, right ventral colon (RVC), left ventral colon (LVC), left dorsal colon (LDC), right dorsal colon (RDC), small colon and faeces demonstrated as a proportion of the total number of O.T.U.’S identified in each gut region. Four different clustering cut offs; 90%, 95%, 97% and 99% are shown. Core community is defined as those OTUs present in all ten animals and which abundances are 0.1% (or greater) of the total number of sequences in that gut region.

**Figure 4:**
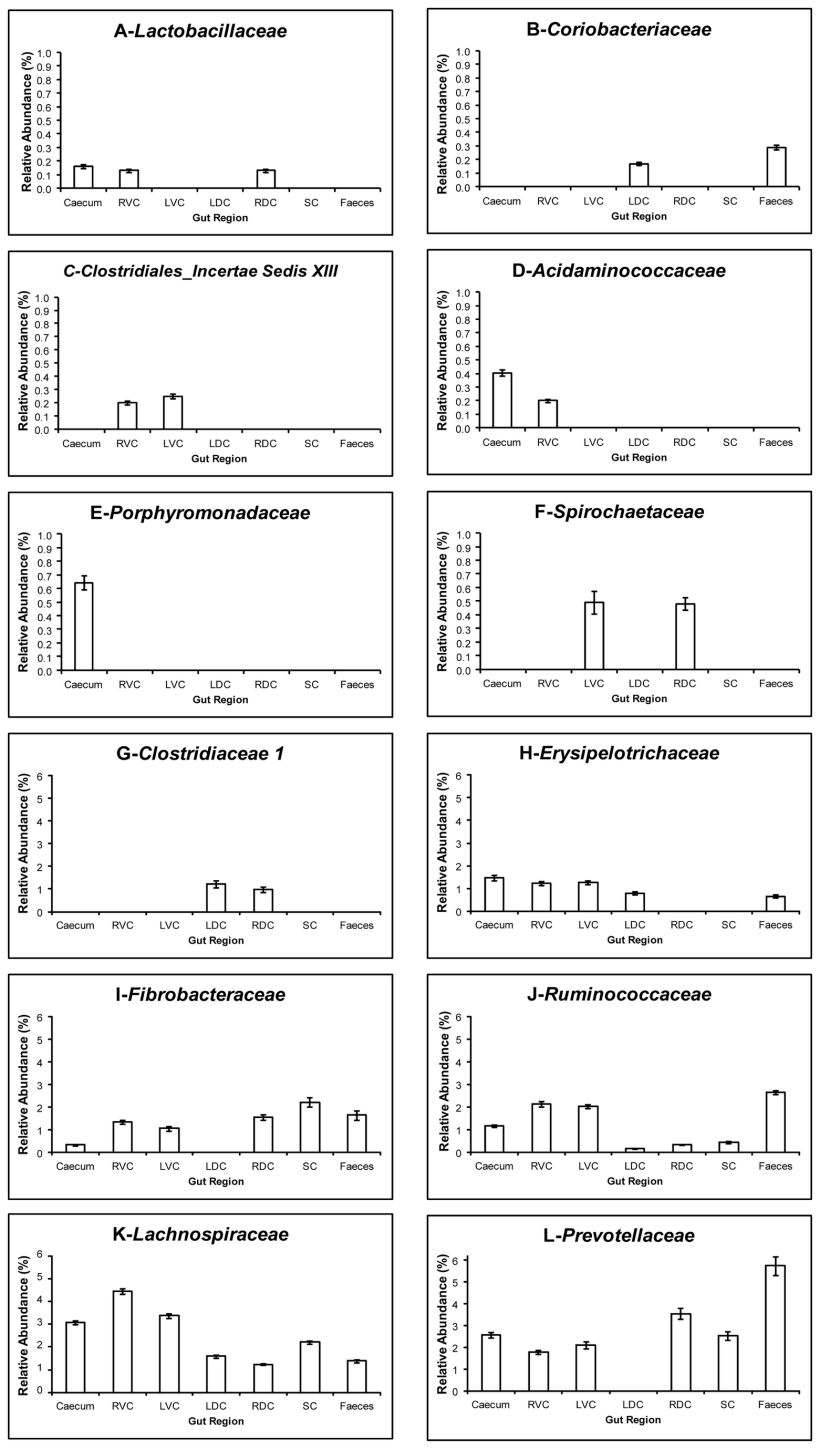
Relative abundance of the most prevalent bacterial families identified as the core bacterial community. Regions of the large intestine included are the caecum, right ventral colon (RVC), left ventral colon (LVC), left dorsal colon (LDC), right dorsal colon (RDC), small colon and faeces. Values presented do not account all for members of stated families found in each gut region only those identified as being part of the core; error bars represent the standard deviation. A-*Lactobacillaceae* B-*Clostridiales*_*Insertae* *Sedis* *XIII* **C**- *Coriobacteriaceae* D-*Acidaminococcacaea* E-*Porphyromondaceae* F-*Spirochaetaceae* G-*Clostridiaceae 1* H*-Erysipelotrichacea* I-*Fibrobacteraceae* J-*Ruminococcaceae* K-*Lachnospiraceae* L-*Prevotellaceae*.

When classified to family level (See Table 3) it can be seen that the core community in the ileum is dominated by *Lactobacillaceae* a member of the *Firmicutes* phyla (16.4%) followed by *Pasteurellaceae* belonging to the phyla *Proteobacteria* (8.57%) with only four families making up the core. In the regions of the large intestine the core is comprised of more families (from 6 in the SC to 11 in the RVC & RDC) and the dominant families change in a similar way to the pattern described previously in Figure 2. The core in the regions of the proximal large intestine (Caecum, RVC & LVC) is dominated (in order of largest to smallest abundance) by an unclassified family belonging to the order *Bacteroidales*, the family *Lachnospiraceae* (*Firmicutes* phyla), *Prevotellaceae* (*Bacteroidetes* phyla), *Erysipelotrichaceae* (*Firmicutes* phyla), *Ruminococcaceae* (*Firmicutes* phyla) and *Fibrobacteraceae* (*Fibrobacteres* phyla). The core in the regions of the distal large intestine (RDC , SC & faeces) is then dominated by (in order of largest to smallest abundance) *Prevotellaceae, Fibrobacteraceae, Lachnospiraceae*, unclassified family only classified to phyla level as *Bacteroidetes* and *Clostridiaceae 1* (Firmicutes phyla). The LDC is dominated by the *Lachnospiraceae, Clostridiaceae 1*, unclassified family belonging to the order *Bacteroidales* and *Erysipelotrichaceae*. The way in which the bacterial families representing the core bacterial community in the large intestine change in terms of relative abundance through the gut can be seen in Figure 4. This clearly illustrates the distinct change in the bacterial community between the ileum and the hind gut and at the pelvic flexure separating the proximal and distal large intestine. 

**Table 3:** Classification of the core bacterial community in different compartments of the horse’s large intestine.

	**O.T.U. numbers**	**Phylum**	**Class**	**Order**	**Family**	**Relative Abundance %**	**Standard Deviation**
**ILEUM**	19	Firmicutes	Clostridia	Clostridiales	Clostridiaceae 1	3.32	0.637
	18;314	Firmicutes	Bacilli	Lactobacillales	Streptococcaceae	3.77	1.555
	9;23	Proteobacteria	Gammaproteobacteria	Pasteurellales	Pasteurellaceae	8.47	0.813
	1;3	Firmicutes	Bacilli	Lactobacillales	Lactobacillaceae	16.35	0.462
**CAECUM**	1	Firmicutes	Bacilli	Lactobacillales	Lactobacillaceae	0.16	0.015
	40	Unclassified	Unclassified	Unclassified	Unclassified	0.33	0.022
	66	Fibrobacteres	Fibrobacteria	Fibrobacterales	Fibrobacteraceae	0.35	0.031
	535	Firmicutes	Negativicutes	Selenomonadales	Acidaminococcaceae	0.41	0.022
	105	Bacteroidetes	Bacteroidia	Bacteroidales	Porphyromonadaceae	0.64	0.052
	52;484;500	Firmicutes	Clostridia	Clostridiales	Ruminococcaceae	1.17	0.049
	16	Firmicutes	Erysipelotrichia	Erysipelotrichales	Erysipelotrichaceae	1.47	0.127
	32;55;139;485;524;960	Bacteroidetes	Bacteroidia	Bacteroidales	Prevotellaceae	2.57	0.127
	78;118;125;237;390;670;687;2050	Firmicutes	Clostridia	Clostridiales	Lachnospiraceae	3.09	0.087
	82;115;146;158;216;278;495;655	Bacteroidetes	Bacteroidia	Bacteroidales	unclassified	3.74	0.194
**RVC**	860	Firmicutes	Clostridia	Clostridiales	unclassified	0.11	0.009
	1	Firmicutes	Bacilli	Lactobacillales	Lactobacillaceae	0.13	0.009
	147	Firmicutes	Clostridia	Clostridiales	Clostridiales_Incertae Sedis XIII	0.20	0.017
	535	Firmicutes	Negativicutes	Selenomonadales	Acidaminococcaceae	0.20	0.017
	40	unclassified	unclassified	unclassified	unclassified	0.65	0.043
	16	Firmicutes	Erysipelotrichia	Erysipelotrichales	Erysipelotrichaceae	1.24	0.081
	39;66	Fibrobacteres	Fibrobacteria	Fibrobacterales	Fibrobacteraceae	1.36	0.089
	32;55	Bacteroidetes	Bacteroidia	Bacteroidales	Prevotellaceae	1.77	0.095
	52;93;207;484;500	Firmicutes	Clostridia	Clostridiales	Ruminococcaceae	2.12	0.114
	82;155;216;490;497;529	Bacteroidetes	Bacteroidia	Bacteroidales	unclassified	2.36	0.118
	6;78;118;125;143;149;193;262;265;288;390;999	Firmicutes	Clostridia	Clostridiales	Lachnospiraceae	4.45	0.127
**LVC**	302	Firmicutes	Clostridia	Clostridiales	Clostridiales_Incertae Sedis XII	0.25	0.019
	50	Spirochaetes	Spirochaetes	Spirochaetales	Spirochaetaceae	0.49	0.081
	40	unclassified	unclassified	unclassified	unclassified	0.77	0.041
	39;66	Fibrobacteres	Fibrobacteria	Fibrobacterales	Fibrobacteraceae	1.07	0.102
	16	Firmicutes	Erysipelotrichia	Erysipelotrichales	Erysipelotrichaceae	1.26	0.088
	52;73;93;236;484;1529	Firmicutes	Clostridia	Clostridiales	Ruminococcaceae	2.02	0.081
	32;55	Bacteroidetes	Bacteroidia	Bacteroidales	Prevotellaceae	2.09	0.161
	82;140;155;158;476;495	Bacteroidetes	Bacteroidia	Bacteroidales	unclassified	2.93	0.120
	61;118;149;193;237;288;390;431	Firmicutes	Clostridia	Clostridiales	Lachnospiraceae	3.38	0.120
**LDC**	201	Actinobacteria	Actinobacteria	Coriobacteriales	Coriobacteriaceae	0.17	0.009
	207	Firmicutes	Clostridia	Clostridiales	Ruminococcaceae	0.18	0.015
	46	Bacteroidetes	unclassified	unclassified	unclassified	0.63	0.140
	16	Firmicutes	Erysipelotrichia	Erysipelotrichales	Erysipelotrichaceae	0.79	0.061
	82	Bacteroidetes	Bacteroidia	Bacteroidales	unclassified	0.80	0.101
	72	Firmicutes	Clostridia	Clostridiales	Clostridiaceae 1	1.21	0.160
	38;61;78;149;288;340	Firmicutes	Clostridia	Clostridiales	Lachnospiraceae	1.59	0.079
**RDC**	7	Firmicutes	Bacilli	Lactobacillales	Lactobacillaceae	0.13	0.009
	69	Firmicutes	Clostridia	Clostridiales	Ruminococcaceae	0.34	0.019
	95	Spirochaetes	Spirochaetes	Spirochaetales	Spirochaetaceae	0.48	0.046
	35	unclassified	unclassified	unclassified	unclassified	0.59	0.051
	17	Firmicutes	unclassified	unclassified	unclassified	0.72	0.154
	82;158;495	Bacteroidetes	Bacteroidia	Bacteroidales	unclassified	0.98	0.066
	19;72	Firmicutes	Clostridia	Clostridiales	Clostridiaceae 1	0.98	0.115
	46	Bacteroidetes	unclassified	unclassified	unclassified	1.04	0.196
	38;118;149	Firmicutes	Clostridia	Clostridiales	Lachnospiraceae	1.24	0.046
	11	Fibrobacteres	Fibrobacteria	Fibrobacterales	Fibrobacteraceae	1.56	0.112
	8;14;32	Bacteroidetes	Bacteroidia	Bacteroidales	Prevotellaceae	3.55	0.250
**SC**	82	Bacteroidetes	Bacteroidia	Bacteroidales	Unclassified	0.44	0.050
	35	Unclassified	Unclassified	Unclassified	Unclassified	0.45	0.034
	69;191;263;273;371	Firmicutes	Clostridia	Clostridiales	Ruminococcaceae	1.22	0.059
	22;38;118;288;365	Firmicutes	Clostridia	Clostridiales	Lachnospiraceae	2.21	0.077
	11	Fibrobacteres	Fibrobacteria	Fibrobacterales	Fibrobacteraceae	2.23	0.195
	14;32;55	Bacteroidetes	Bacteroidia	Bacteroidales	Prevotellaceae	2.54	0.199
**FAECES**	127	Bacteroidetes	Sphingobacteria	Sphingobacteriales	Unclassified	0.27	0.021
	132;201	Actinobacteria	Actinobacteria	Coriobacteriales	Coriobacteriaceae	0.29	0.018
	82	Bacteroidetes	Bacteroidia	Bacteroidales	Unclassified	0.33	0.029
	16	Firmicutes	Erysipelotrichia	Erysipelotrichales	Erysipelotrichaceae	0.66	0.055
	35;111	Unclassified	Unclassified	Unclassified	Unclassified	0.72	0.038
	38;143;149	Firmicutes	Clostridia	Clostridiales	Lachnospiraceae	1.39	0.068
	11	Fibrobacteres	Fibrobacteria	Fibrobacterales	Fibrobacteraceae	1.65	0.200
	63;69;97;122;191;207;236;240;273;415	Firmicutes	Clostridia	Clostridiales	Ruminococcaceae	2.64	0.088
	8;14;21;32	Bacteroidetes	Bacteroidia	Bacteroidales	Prevotellaceae	5.74	0.417

Data shown from the ileum, caecum, right ventral colon (RVC), left ventral colon (LVC), left dorsal colon (LDC), right dorsal colon (RDC), small colon and faeces classified to family level. Core community is defined as those OTUs (clustered at 97% similarity) present in all ten animals and which abundances are 0.1% (or greater) of the total number of sequences in that gut region OTUs are grouped by family and the relative abundance of each family is given as a percentage of the total number of sequences for each gut region.

## Discussion

The herbivore gastrointestinal tract harbors an abundant and diverse microbial community which provides the host with essential nutrients through degradation and fermentation of complex carbohydrates from the diet that would otherwise be unavailable [38,39]. In addition, to this primary function, the microbial community serves the host by modulating immune function and maintaining health [40]. The horse receives much of its dietary energy through hydrolysis and fermentation of fibre in the large intestine which comprises of large chambers including the caecum and the colon. In these regions the microbiota ferments fibre to yield volatile fatty acids and lactic acid which can be absorbed and utilised as energy sources [12-14]. Despite the importance to the horse of fermentation in the large intestine this system is easily perturbed. The largest factor influencing the microbiota is the diet of the animal; work by Ley and colleagues [39] demonstrates phylogenetic separation of carnivores, omnivores and herbivores. In the horse differences in bacterial number, composition and activity are known to occur when feeding a fibre based compared to a starch rich diet [22,41,42]. 

In previous work we have demonstrated that there are differences in the microbial community found in the caecum compared to the RDC and faeces of horses and ponies [37]; this has been reinforced by the current work. This change coincides physiologically with the pelvic flexure between the LVC and LDC where the intestine significantly narrows and folds back on itself. This anatomical feature has a marked effect on digesta passage and is one of the most common locations for impaction colic in the horse [43,44]. Here we show, similar to the human gut [45], that the bacterial diversity in the ileum of the equid is lower than that found in the large intestine. The diversity and species richness in the large intestine increases from the caecum to be highest in the RVC before declining through the remainder of the gut to the faeces, confirming our previous findings [37]. 

*Firmicutes* and *Bacteroidetes* tend to be the predominant phyla in most mammalian gut environments studied to date [38,46,47]. A higher ratio of *Bacteroidetes* to *Firmicutes* has been linked with conditions such as obesity [48] and IBD [49]. In the human colon and faeces the microbiota is approximately 50-95% Firmicutes, 3-50% *Bacteroidetes* and 1-3% *Actinobacteria* [4,38,50,51]. In the horse published work utilising next generation sequencing is only starting to appear and is limited to a few studies, using faeces, that are of variable quality in terms of sample replication and sequencing depth [28,29,52]. From these early studies the dominant phylum in the horse appears to be *Firmicutes* (43-69%), with inconsistency over the next most abundant (*Bacteroidetes* 14.2%, *Proteobacteria* 10.2% [29], *Proteobacteria* and *Verrucomicrobia* 4.1% each, *Bacteroidetes* 3.65% [52] or *Verrucomicrobia* 18.1%, *Bacteroidetes* 5.7% [28], with *Spirochaetes* and *Actinobacteria* also identified. Older culture independent work that utilised cloning or probe based methods describe a similar profile [53-55] but with much higher levels of *Bacteroidetes* (45-49%) identified in some studies [55,56]. Our work identifies a phyla profile (from animals on a grass based diet) that is reasonably consistent across all regions of the large intestine with the most dominant phylum being *Firmicutes* (average 46%), *Bacteroidetes* (average 43%) followed by *Fibrobacteres, Spirochaetes* and *Proteobacteria* (all <4%) with a notable lack of *Verrucomicrobia* (similar to Costa and colleagues) [29]. We also found that the ileum, although still dominated by the *Firmicutes* (70%), had a high abundance of *Proteobacteria* (14%) and a lower amount of *Bacteroidetes* (10%); a pattern shown in the human ileum [45,57] and in the gastric mucosa of the horse [58].

The presence of a core microbial community in the human gut has been identified but it differs in size dependent on the study design. A shared core was only identified in 50% of individuals [51] whereas Hamady and Knight [59] proposed that a core may exist within sub-communities rather than in the entire community. Another suggestion was that a core may not exist as a phylogenetic one but at a functional level [1]. More recently core species were identified but at massive sequencing depth or by using different methods of OTU clustering and classification [2,3,60]. What is clear is that what constitutes a core microbiome has not been well defined. The sequencing depth used may restrict identification of the presence of a core and thus the true size of that core, similarly OTU clustering methods may influence detection of core members [3,4,60]. Although varying numbers of phylotypes/ species/ OTUs have been identified as potentially belonging to the core community in the human there is a common thread identifying Clostridia as the most prevalent class of bacteria in the core [2-4,45,51]. Members of this class identified as being part of the core are *Ruminococcaceae, Lachnospiraceae, Clostridiaceae, Streptococcaceae* [2-4,51]. Bacteroidetes have also been shown to be core but at low levels [4,51]. *Lachnospiraceae* in particular have been shown to exist in most samples [2-4]. When Sekelja and colleagues [3] analysed sequence data from Ley and others [8] they found this family across many mammals including 71% of the order Perissodactyla included in the study suggesting this may appear in the core of most mammals. Interestingly low levels of this family in the human gut have been implicated in IBD [49]. This role is perhaps not surprising given that *Lachnospiraceae* are prolific producers of butyrate [4,61] and butyrate is known to have a protective function on colonocytes in the gut wall [62,63]. 

To the authors’ knowledge only one study has commented on the possibility of a core bacterial community in the horse gut [29]. They identified 123 shared OTUs from the faeces of 4 horses (out of 1620 identified in total) but of those only 6 had an abundance of greater than 25 occurrences per animal. They classified 5 of these OTUs as *Lachnospiraceae* with the remaining one being unclassified. This is larger than the core that we have identified in faeces (25/2566 in this study) however we have selected only those occurring at least once in every animal and at 0.1% or greater of the total sequences, so if treated in a similar way to Costa and others [29] it is likely that a higher number of OTUs would have been ‘core’. This again highlights the lack of a universal definition as to what constitutes a core. A study in cattle of the core community identified from faecal samples was dominated by *Prevotella* spp followed by the *Lachnospiraceae* family, *Faecalibacterium* spp then *Ruminococcus* spp [64]. The most abundant family we identified as belonging to the core in equid faeces was *Prevotellaceae* followed by *Ruminoccocaceae*, *Fibrobacteraceae* then *Lachnospiraceae* which is similar to what was reported in cattle [64,65] and we also noted a high amount of *Clostridia* similar to the human literature described earlier. The fact that both our work and that of Costa and others [29] identified high levels of *Lachnospiraceae* would seem sensible given the work of Sekelja and others [3], discussed earlier. Unfortunately, as Costa and colleagues [29] do not state what all of the shared OTUs were classified as, it is not possible to compare this with the prevalence of the other families we identified as part of the core. As the most dominant core bacteria change through the large intestine it would be of interest to investigate their functional significance as to whether the role these bacteria play alter in a similar way .

We have shown for the first time that a phylogenetic core bacterial community exists in all regions of the large intestine of healthy horses on a fibre based, grass diet. This core community is smaller than found in the rumen of the cow [65] but unlike most other core communities that have been identified from other environments is not dominated by any particular OTUs. In the oral microbiota of both the dog and human a small core is seen but with several highly abundant OTUs [66,67] and in the porcine tonsils a larger core was observed but again with a few highly dominant OTUs [68]. These other environments are notably less susceptible to disturbance than the gut of the horse and as such the fact they appear to have ‘key’ members of their core which are highly prevalent (compared to the horse hindgut where the core is made up of many low abundance OTUs) may suggest an explanation as to why the equine hind gut is so vulnerable to change when challenged leading to an alteration in fermentation patterns and consequent metabolic disorders. 

Further consideration must now be given to the functional role of these bacteria in order to establish how much functional redundancy exists and which bacteria are essential to maintain normal digestive function and health. By knowing what makes up the core community comparisons can now be made to different diets and when metabolic disorders (such as Laminitis and colic) are encountered allowing suggestion to be made regarding prophylaxis, diagnosis and treatment.

## Supporting Information

Figure S1 **Rarefaction Curves showing depth of sequencing of the microbial communities in the horse’s Ileum, caecum, right ventral colon (RVC), left ventral colon (LVC), left dorsal colon (LDC), right dorsal colon (RDC), small colon and faeces (Calculated from non-normalised data and the average of each gut region across all animals).**
(DOCX)

Figure S2 **Good’s Coverage Estimates showing depth of sequencing of the microbial communities in the horse’s Ileum, caecum, right ventral colon (RVC), left ventral colon (LVC), left dorsal colon (LDC), right dorsal colon (RDC), small colon and faeces calculated by gut region (error bars show standard deviation).**
(DOCX)

Figure S3 **Good’s Coverage Estimates showing depth of sequencing of the microbial communities in the horse’s Ileum, caecum, right ventral colon (RVC), left ventral colon (LVC), left dorsal colon (LDC), right dorsal colon (RDC), small colon and faeces calculated by animal (error bars show standard deviation).**
(DOCX)

Table S1 **Animal metadata.**
(DOCX)

Table S2 **MID barcode sequences used for multiplexed 454 pyrosequencing.**
(DOCX)

Table S3 **The relative abundance (%) of the core bacterial community in different compartments of the horse’s large intestine, further information for Figure 3.**
(DOCX)
