# Supplementary material for: Identification of a Core Bacterial Community within the Large Intestine of the Horse
Source: PLoS One. 2013 Oct 24;8(10):e77660. doi: 10.1371/journal.pone.0077660 (PMC3812009; doi:10.1371/journal.pone.0077660)

Figure S1- Rarefaction Curves showing depth of sequencing of the microbial communities in the horse’s Ileum, caecum, right ventral colon (RVC), left ventral colon (LVC), left dorsal colon (LDC), right dorsal colon (RDC), small colon and faeces (Calculated from non-normalised data and the average of each gut region across all animals)


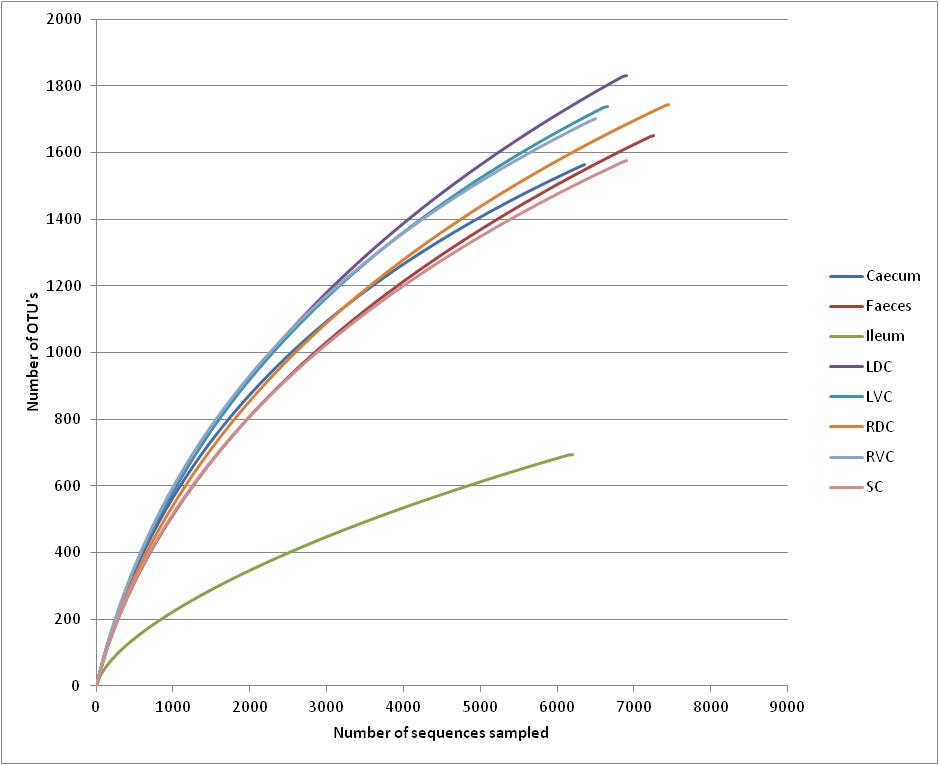

Supplement: Figure S1 — Rarefaction Curves showing depth of sequencing of the microbial communities in the horse’s Ileum, caecum, right ventral colon (RVC), left ventral colon (LVC), left dorsal colon (LDC), right dorsal colon (RDC), small colon and faeces (Calculated from non-normalised data and the average of each gut region across all animals). (DOCX) [file pone.0077660.s001.docx]
