# Supplementary material for: Identification of a Core Bacterial Community within the Large Intestine of the Horse
Source: PLoS One. 2013 Oct 24;8(10):e77660. doi: 10.1371/journal.pone.0077660 (PMC3812009; doi:10.1371/journal.pone.0077660)

Figure S2- Good’s Coverage Estimates showing depth of sequencing of the microbial communities in the horse’s Ileum, caecum, right ventral colon (RVC), left ventral colon (LVC), left dorsal colon (LDC), right dorsal colon (RDC), small colon and faeces calculated by gut region( error bars show standard deviation)


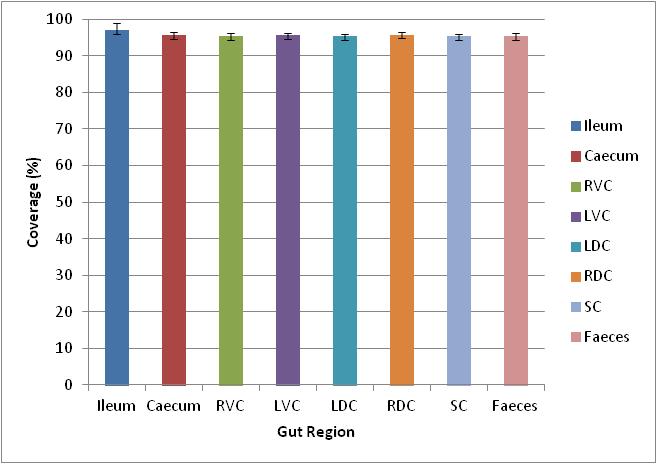

Supplement: Figure S2 — Good’s Coverage Estimates showing depth of sequencing of the microbial communities in the horse’s Ileum, caecum, right ventral colon (RVC), left ventral colon (LVC), left dorsal colon (LDC), right dorsal colon (RDC), small colon and faeces calculated by gut region (error bars show standard deviation). (DOCX) [file pone.0077660.s002.docx]
