# Supplementary material for: Identification of a Core Bacterial Community within the Large Intestine of the Horse
Source: PLoS One. 2013 Oct 24;8(10):e77660. doi: 10.1371/journal.pone.0077660 (PMC3812009; doi:10.1371/journal.pone.0077660)

Figure S3- Good’s Coverage Estimates showing depth of sequencing of the microbial communities in the horse’s Ileum, caecum, right ventral colon (RVC), left ventral colon (LVC), left dorsal colon (LDC), right dorsal colon (RDC), small colon and faeces calculated by animal( error bars show standard deviation)


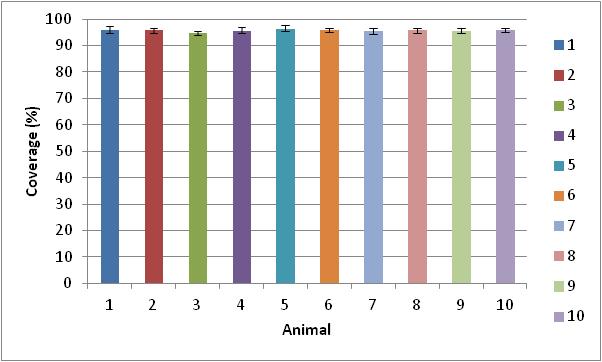

Supplement: Figure S3 — Good’s Coverage Estimates showing depth of sequencing of the microbial communities in the horse’s Ileum, caecum, right ventral colon (RVC), left ventral colon (LVC), left dorsal colon (LDC), right dorsal colon (RDC), small colon and faeces calculated by animal (error bars show standard deviation). (DOCX) [file pone.0077660.s003.docx]
