# Supplementary material for: Identification of a Core Bacterial Community within the Large Intestine of the Horse
Source: PLoS One. 2013 Oct 24;8(10):e77660. doi: 10.1371/journal.pone.0077660 (PMC3812009; doi:10.1371/journal.pone.0077660)
Supplement: Table S1 — Animal metadata. (DOCX) [file pone.0077660.s004.docx]

Table S1- Animal metadata

| **Horse** | **Age** | **Breed** | **Sex** | **Height (approx)** | **Diet** | **Distance travelled (miles)** | **Health status inc. Body condition score (BCS)*** |
| --- | --- | --- | --- | --- | --- | --- | --- |
| **1** | 8 | Thoroughbred | Gelding | 16.2hh | Grass  (hay 12 hours euthanasia) | 80 | No known issues  BCS- 2 |
| **2** | 8 | Thoroughbred | Gelding | 16.2hh | Grass  (hay 12 hours pre-euthanasia) | 80 | No known issues  BCS- 2 |
| **3** | 9 | Thoroughbred | Gelding | 16.2hh | Grass  (hay 12 hours pre-euthanasia) | 80 | No known issues  BCS- 2 |
| **4** | 7 | Thoroughbred | Gelding | 16.2hh | Grass  (hay 12 hours pre-euthanasia) | 80 | No known issues  BCS- 2 |
| **5** | 5 | Thoroughbred | Mare | 16.2hh | Grass  (hay 12 hours pre-euthanasia) | 80 | No known issues  BCS- 2 |
| **6** | 17 | New Forest pony | Mare | 13hh | Grass  (hay 12 hours pre-euthanasia) | 90 | No known issues  BCS- 2.5 |
| **7** | 13 | New Forest pony | Mare | 13hh | Grass  (hay 12 hours pre-euthanasia) | 90 | No known issues  BCS- 2.5 |
| **8** | 19 | New Forest pony | Mare | 13hh | Grass  (hay 12 hours pre-euthanasia) | 90 | No known issues  BCS- 2.5 |
| **9** | 7 | New Forest pony | Mare | 13hh | Grass  (hay 12 hours pre-euthanasia) | 90 | No known issues  BCS- 2.5 |
| **10** | 8 | New Forest pony | Mare | 13hh | Grass  (hay 12 hours pre-euthanasia) | 90 | No known issues  BCS- 2.5 |

* Body condition score estimated using the method of Carroll and Huntington, 1988
