# Supplementary material for: Identification of a Core Bacterial Community within the Large Intestine of the Horse
Source: PLoS One. 2013 Oct 24;8(10):e77660. doi: 10.1371/journal.pone.0077660 (PMC3812009; doi:10.1371/journal.pone.0077660)
Supplement: Table S2 — MID barcode sequences used for multiplexed 454 pyrosequencing. (DOCX) [file pone.0077660.s005.docx]

Table S2- MID barcode sequences used for multiplexed 454 pyrosequencing

| **MID number** | **Sequence** | **MID number2** | **Sequence3** |
| --- | --- | --- | --- |
| MID-1 | ACGAGTGCGT | MID-23 | TACTCTCGTG |
| MID-2 | ACGCTCGACA | MID-24 | TAGAGACGAG |
| MID-3 | AGACGCACTC | MID-25 | TCGTCGCTCG |
| MID-4 | AGCACTGTAG | MID-26 | ACATACGCGT |
| MID-5 | ATCAGACACG | MID-27 | ACGCGAGTAT |
| MID-6 | ATATCGCGAG | MID-28 | ACTACTATGT |
| MID-7 | CGTGTCTCTA | MID-29 | ACTGTACAGT |
| MID-8 | CTCGCGTGTC | MID-30 | AGACTATACT |
| MID-10 | TCTCTATGCG | MID-31 | AGCGTCGTCT |
| MID-11 | TGATACGTCT | MID-32 | AGTACGCTAT |
| MID-13 | CATAGTAGTG | MID-33 | ATAGAGTACT |
| MID-14 | CGAGAGATAC | MID-34 | CACGCTACGT |
| MID-15 | ATACGACGTA | MID-35 | CAGTAGACGT |
| MID-16 | TCACGTACTA | MID-36 | CGACGTGACT |
| MID-17 | CGTCTAGTAC | MID-37 | TACACACACT |
| MID-18 | TCTACGTAGC | MID-38 | TACACGTGAT |
| MID-19 | TGTACTACTC | MID-39 | TACAGATCGT |
| MID-20 | ACGACTACAG | MID-40 | TACGCTGTCT |
| MID-21 | CGTAGACTAG | MID-41 | TAGTGTAGAT |
| MID-22 | TACGAGTATG | MID-42 | TCGATCACGT |
