# Supplementary material for: Identification of a Core Bacterial Community within the Large Intestine of the Horse
Source: PLoS One. 2013 Oct 24;8(10):e77660. doi: 10.1371/journal.pone.0077660 (PMC3812009; doi:10.1371/journal.pone.0077660)
Supplement: Table S3 — The relative abundance (%) of the core bacterial community in different compartments of the horse’s large intestine, further information for Figure 3. (DOCX) [file pone.0077660.s006.docx]

**Table S3- The relative abundance (%) of the core bacterial community in different compartments of the horse’s large intestine, further information for figure 3.**

| **O.T.U.** | **Ileum** | **Caecum** | **RVC** | **LVC** | **LDC** | **RDC** | **SC** | **Faeces** | **Phyla** | **Class** | **Order** | **Family** |
| --- | --- | --- | --- | --- | --- | --- | --- | --- | --- | --- | --- | --- |
| 1 | 10.93 | 0.16 | 0.13 |  |  |  |  |  | Firmicutes | Bacilli | Lactobacillales | Lactobacillaceae |
| 3 | 5.43 |  |  |  |  |  |  |  | Firmicutes | Bacilli | Lactobacillales | Lactobacillaceae |
| 7 |  |  |  |  |  | 0.13 |  |  | Firmicutes | Bacilli | Lactobacillales | Lactobacillaceae |
| 8 |  |  |  |  |  | 1.70 |  | 2.41 | Bacteroidetes | Bacteroidia | Bacteroidales | Prevotellaceae |
| 9 | 4.47 |  |  |  |  |  |  |  | Proteobacteria | Gammaproteobacteria | Pasteurellales | Pasteurellaceae |
| 11 |  |  |  |  |  | 1.56 | 2.23 | 1.65 | Fibrobacteres | Fibrobacteria | Fibrobacterales | Fibrobacteraceae |
| 14 |  |  |  |  |  | 1.21 | 1.75 | 1.99 | Bacteroidetes | Bacteroidia | Bacteroidales | Prevotellaceae |
| 16 |  | 1.47 | 1.24 | 1.26 | 0.79 |  |  | 0.66 | Firmicutes | Erysipelotrichia | Erysipelotrichales | Erysipelotrichaceae |
| 17 |  |  |  |  |  | 0.72 |  |  | Firmicutes | unclassified | unclassified | unclassified |
| 18 | 3.20 |  |  |  |  |  |  |  | Firmicutes | Bacilli | Lactobacillales | Streptococcaceae |
| 19 | 3.32 |  |  |  |  | 0.46 |  |  | Firmicutes | Clostridia | Clostridiales | Clostridiaceae 1 |
| 21 |  |  |  |  |  |  |  | 1.05 | Bacteroidetes | Bacteroidia | Bacteroidales | Prevotellaceae |
| 22 |  |  |  |  |  |  | 0.55 |  | Firmicutes | Clostridia | Clostridiales | Lachnospiraceae |
| 23 | 4.00 |  |  |  |  |  |  |  | Proteobacteria | Gammaproteobacteria | Pasteurellales | Pasteurellaceae |
| 32 |  | 0.93 | 1.10 | 1.13 |  | 0.64 | 0.53 | 0.28 | Bacteroidetes | Bacteroidia | Bacteroidales | Prevotellaceae |
| 35 |  |  |  |  |  | 0.59 | 0.45 | 0.51 | Unclassified | Unclassified | Unclassified | Unclassified |
| 38 |  |  |  |  | 0.29 | 0.65 | 0.94 | 1.05 | Firmicutes | Clostridia | Clostridiales | Lachnospiraceae |
| 39 |  |  | 0.85 | 0.64 |  |  |  |  | Fibrobacteres | Fibrobacteria | Fibrobacterales | Fibrobacteraceae |
| 40 |  | 0.33 | 0.65 | 0.77 |  |  |  |  | Unclassified | Unclassified | Unclassified | Unclassified |
| 46 |  |  |  |  | 0.63 | 1.04 |  |  | Bacteroidetes | unclassified | unclassified | unclassified |
| 50 |  |  |  | 0.49 |  |  |  |  | Spirochaetes | Spirochaetes | Spirochaetales | Spirochaetaceae |
| 52 |  | 0.60 | 0.65 | 0.47 |  |  |  |  | Firmicutes | Clostridia | Clostridiales | Ruminococcaceae |
| 55 |  | 0.77 | 0.68 | 0.96 |  |  | 0.26 |  | Bacteroidetes | Bacteroidia | Bacteroidales | Prevotellaceae |
| 61 |  |  | 0.73 | 0.85 | 0.38 |  |  |  | Firmicutes | Clostridia | Clostridiales | Lachnospiraceae |
| 63 |  |  |  |  |  |  |  | 0.42 | Firmicutes | Clostridia | Clostridiales | Ruminococcaceae |
| 66 |  | 0.35 | 0.51 | 0.43 |  |  |  |  | Fibrobacteres | Fibrobacteria | Fibrobacterales | Fibrobacteraceae |
| 69 |  |  |  |  |  | 0.34 | 0.51 | 0.52 | Firmicutes | Clostridia | Clostridiales | Ruminococcaceae |
| 72 |  |  |  |  | 1.21 | 0.52 |  |  | Firmicutes | Clostridia | Clostridiales | Clostridiaceae 1 |
| 73 |  |  |  | 0.27 |  |  |  |  | Firmicutes | Clostridia | Clostridiales | Ruminococcaceae |
| 78 |  | 0.43 | 0.45 |  | 0.20 |  |  |  | Firmicutes | Clostridia | Clostridiales | Lachnospiraceae |
| 82 |  | 1.18 | 1.15 | 1.10 | 0.80 | 0.51 | 0.44 | 0.33 | Bacteroidetes | Bacteroidia | Bacteroidales | unclassified |
| 93 |  |  | 0.63 | 0.51 |  |  |  |  | Firmicutes | Clostridia | Clostridiales | Ruminococcaceae |
| 95 |  |  |  |  |  | 0.48 |  |  | Spirochaetes | Spirochaetes | Spirochaetales | Spirochaetaceae |
| 97 |  |  |  |  |  |  |  | 0.24 | Firmicutes | Clostridia | Clostridiales | Ruminococcaceae |
| 105 |  | 0.64 |  |  |  |  |  |  | Bacteroidetes | Bacteroidia | Bacteroidales | Porphyromonadaceae |
| 111 |  |  |  |  |  |  |  | 0.21 | Unclassified | Unclassified | Unclassified | Unclassified |
| 115 |  | 0.55 |  |  |  |  |  |  | Bacteroidetes | Bacteroidia | Bacteroidales | unclassified |
| 118 |  | 0.57 | 0.56 | 0.68 |  | 0.40 | 0.35 |  | Firmicutes | Clostridia | Clostridiales | Lachnospiraceae |
| 122 |  |  |  |  |  |  |  | 0.35 | Firmicutes | Clostridia | Clostridiales | Ruminococcaceae |
| 125 |  | 0.79 | 0.62 |  |  |  |  |  | Firmicutes | Clostridia | Clostridiales | Lachnospiraceae |
| 127 |  |  |  |  |  |  |  | 0.27 | Bacteroidetes | Sphingobacteria | Sphingobacteriales | Unclassified |
| 132 |  |  |  |  |  |  |  | 0.15 | Actinobacteria | Actinobacteria | Coriobacteriales | Coriobacteriaceae |
| 139 |  | 0.29 |  |  |  |  |  |  | Bacteroidetes | Bacteroidia | Bacteroidales | Prevotellaceae |
| 140 |  |  |  | 0.38 |  |  |  |  | Bacteroidetes | Bacteroidia | Bacteroidales | unclassified |
| 143 |  |  | 0.25 |  |  |  |  | 0.12 | Firmicutes | Clostridia | Clostridiales | Lachnospiraceae |
| 146 |  | 0.61 |  |  |  |  |  |  | Bacteroidetes | Bacteroidia | Bacteroidales | unclassified |
| 147 |  |  | 0.20 |  |  |  |  |  | Firmicutes | Clostridia | Clostridiales | Clostridiales_Incertae Sedis XIII |
| 149 |  |  | 0.53 | 0.47 | 0.41 | 0.19 |  | 0.21 | Firmicutes | Clostridia | Clostridiales | Lachnospiraceae |
| 155 |  |  | 0.11 | 0.15 |  |  |  |  | Bacteroidetes | Bacteroidia | Bacteroidales | unclassified |
| 158 |  | 0.20 |  | 0.69 |  | 0.27 |  |  | Bacteroidetes | Bacteroidia | Bacteroidales | unclassified |
| 191 |  |  |  |  |  |  | 0.16 | 0.19 | Firmicutes | Clostridia | Clostridiales | Ruminococcaceae |
| 193 |  |  | 0.17 | 0.22 |  |  |  |  | Firmicutes | Clostridia | Clostridiales | Lachnospiraceae |
| 201 |  |  |  |  | 0.17 |  |  | 0.15 | Actinobacteria | Actinobacteria | Coriobacteriales | Coriobacteriaceae |
| 207 |  |  | 0.15 |  | 0.18 |  |  | 0.29 | Firmicutes | Clostridia | Clostridiales | Ruminococcaceae |
| 216 |  | 0.14 | 0.21 |  |  |  |  |  | Bacteroidetes | Bacteroidia | Bacteroidales | unclassified |
| 236 |  |  |  | 0.26 |  |  |  | 0.12 | Firmicutes | Clostridia | Clostridiales | Ruminococcaceae |
| 237 |  | 0.24 |  | 0.25 |  |  |  |  | Firmicutes | Clostridia | Clostridiales | Lachnospiraceae |
| 240 |  |  |  |  |  |  |  | 0.20 | Firmicutes | Clostridia | Clostridiales | Ruminococcaceae |
| 262 |  |  | 0.14 |  |  |  |  |  | Firmicutes | Clostridia | Clostridiales | Lachnospiraceae |
| 263 |  |  |  |  |  |  | 0.11 |  | Firmicutes | Clostridia | Clostridiales | Ruminococcaceae |
| 265 |  |  | 0.16 |  |  |  |  |  | Firmicutes | Clostridia | Clostridiales | Lachnospiraceae |
| 273 |  |  |  |  |  |  | 0.18 | 0.13 | Firmicutes | Clostridia | Clostridiales | Ruminococcaceae |
| 278 |  | 0.46 |  |  |  |  |  |  | Bacteroidetes | Bacteroidia | Bacteroidales | unclassified |
| 288 |  |  | 0.32 | 0.30 | 0.20 |  | 0.27 |  | Firmicutes | Clostridia | Clostridiales | Lachnospiraceae |
| 302 |  |  |  | 0.25 |  |  |  |  | Firmicutes | Clostridia | Clostridiales | Clostridiales_Incertae Sedis XII |
| 314 | 0.57 |  |  |  |  |  |  |  | Firmicutes | Bacilli | Lactobacillales | Streptococcaceae |
| 340 |  |  |  |  | 0.11 |  |  |  | Firmicutes | Clostridia | Clostridiales | Lachnospiraceae |
| 365 |  |  |  |  |  |  | 0.11 |  | Firmicutes | Clostridia | Clostridiales | Lachnospiraceae |
| 371 |  |  |  |  |  |  | 0.25 |  | Firmicutes | Clostridia | Clostridiales | Ruminococcaceae |
| 390 |  | 0.61 | 0.38 | 0.32 |  |  |  |  | Firmicutes | Clostridia | Clostridiales | Lachnospiraceae |
| 415 |  |  |  |  |  |  |  | 0.17 | Firmicutes | Clostridia | Clostridiales | Ruminococcaceae |
| 431 |  |  |  | 0.29 |  |  |  |  | Firmicutes | Clostridia | Clostridiales | Lachnospiraceae |
| 476 |  |  |  | 0.19 |  |  |  |  | Bacteroidetes | Bacteroidia | Bacteroidales | unclassified |
| 484 |  | 0.40 | 0.41 | 0.37 |  |  |  |  | Firmicutes | Clostridia | Clostridiales | Ruminococcaceae |
| 485 |  | 0.22 |  |  |  |  |  |  | Bacteroidetes | Bacteroidia | Bacteroidales | Prevotellaceae |
| 490 |  |  | 0.38 |  |  |  |  |  | Bacteroidetes | Bacteroidia | Bacteroidales | unclassified |
| 495 |  | 0.48 |  | 0.42 |  | 0.19 |  |  | Bacteroidetes | Bacteroidia | Bacteroidales | unclassified |
| 497 |  |  | 0.27 |  |  |  |  |  | Bacteroidetes | Bacteroidia | Bacteroidales | unclassified |
| 500 |  | 0.18 | 0.27 |  |  |  |  |  | Firmicutes | Clostridia | Clostridiales | Ruminococcaceae |
| 524 |  | 0.19 |  |  |  |  |  |  | Bacteroidetes | Bacteroidia | Bacteroidales | Prevotellaceae |
| 529 |  |  | 0.24 |  |  |  |  |  | Bacteroidetes | Bacteroidia | Bacteroidales | unclassified |
| 535 |  | 0.41 | 0.20 |  |  |  |  |  | Firmicutes | Negativicutes | Selenomonadales | Acidaminococcaceae |
| 655 |  | 0.12 |  |  |  |  |  |  | Bacteroidetes | Bacteroidia | Bacteroidales | unclassified |
| 670 |  | 0.10 |  |  |  |  |  |  | Firmicutes | Clostridia | Clostridiales | Lachnospiraceae |
| 687 |  | 0.15 |  |  |  |  |  |  | Firmicutes | Clostridia | Clostridiales | Lachnospiraceae |
| 860 |  |  | 0.11 |  |  |  |  |  | Firmicutes | Clostridia | Clostridiales | unclassified |
| 960 |  | 0.18 |  |  |  |  |  |  | Bacteroidetes | Bacteroidia | Bacteroidales | Prevotellaceae |
| 999 |  |  | 0.14 |  |  |  |  |  | Firmicutes | Clostridia | Clostridiales | Lachnospiraceae |
| 1529 |  |  |  | 0.14 |  |  |  |  | Firmicutes | Clostridia | Clostridiales | Ruminococcaceae |
| 2050 |  | 0.20 |  |  |  |  |  |  | Firmicutes | Clostridia | Clostridiales | Lachnospiraceae |
| Not core | 68.09 | 86.08 | 85.39 | 85.75 | 94.63 | 88.39 | 90.91 | 86.31 |  |  |  |  |
